# Structure and Kinetic Investigation of *Streptococcus pyogenes* Family GH38 α-Mannosidase

**DOI:** 10.1371/journal.pone.0009006

**Published:** 2010-02-03

**Authors:** Michael D. L. Suits, Yanping Zhu, Edward J. Taylor, Julia Walton, David L. Zechel, Harry J. Gilbert, Gideon J. Davies

**Affiliations:** 1 York Structural Biology Laboratory, Department of Chemistry, University of York, York, United Kingdom; 2 Complex Carbohydrate Research Center, University of Georgia, Athens, Georgia, United States of America; 3 Departments of Chemistry, Queen's University, Kingston, Canada; 4 Institute for Cell and Molecular Biosciences, The Medical School, Newcastle University, Newcastle Upon Tyne, United Kingdom; Griffith University, Australia

## Abstract

**Background:**

The enzymatic hydrolysis of α−mannosides is catalyzed by glycoside hydrolases (GH), termed α−mannosidases. These enzymes are found in different GH sequence–based families. Considerable research has probed the role of higher eukaryotic “GH38” α−mannosides that play a key role in the modification and diversification of hybrid *N*-glycans; processes with strong cellular links to cancer and autoimmune disease. The most extensively studied of these enzymes is the *Drosophila* GH38 α−mannosidase II, which has been shown to be a retaining α−mannosidase that targets both α−1,3 and α−1,6 mannosyl linkages, an activity that enables the enzyme to process GlcNAc(Man)_5_(GlcNAc)_2_ hybrid *N*-glycans to GlcNAc(Man)_3_(GlcNAc)_2_. Far less well understood is the observation that many bacterial species, predominantly but not exclusively pathogens and symbionts, also possess putative GH38 α−mannosidases whose activity and specificity is unknown.

**Methodology/Principal Findings:**

Here we show that the *Streptococcus pyogenes* (M1 GAS SF370) GH38 enzyme (Spy1604; hereafter SpGH38) is an α−mannosidase with specificity for α−1,3 mannosidic linkages. The 3D X-ray structure of SpGH38, obtained in native form at 1.9 Å resolution and in complex with the inhibitor swainsonine (*K*_i_ 18 µM) at 2.6 Å, reveals a canonical GH38 five-domain structure in which the catalytic “–1” subsite shows high similarity with the *Drosophila* enzyme, including the catalytic Zn^2+^ ion. In contrast, the “leaving group” subsites of SpGH38 display considerable differences to the higher eukaryotic GH38s; features that contribute to their apparent specificity.

**Conclusions/Significance:**

Although the *in vivo* function of this streptococcal GH38 α−mannosidase remains unknown, it is shown to be an α−mannosidase active on *N*-glycans. SpGH38 lies on an operon that also contains the GH84 hexosaminidase (Spy1600) and an additional putative glycosidase. The activity of SpGH38, together with its genomic context, strongly hints at a function in the degradation of host *N*- or possibly *O*-glycans. The absence of any classical signal peptide further suggests that SpGH38 may be intracellular, perhaps functioning in the subsequent degradation of extracellular host glycans following their initial digestion by secreted glycosidases.

## Introduction

The sugar mannose, particularly α− and β−mannosides, play many and varied roles in biological organisms. β−Mannan is a plant polysaccharide recalcitrant to degradation whereas α−mannans are found in the fungal cell-wall. More subtle roles for α− and β−mannosides are found in the glycans of higher organisms, where oligosaccharide diversity affords cell signaling and recognition events that lead oligosaccharides to play the role of “glycocode”; the language of cellular communication [1]. Furthermore, mannose chemistry itself is extremely challenging, demanding inspired solutions to the problem of synthesis at its occluded anomeric centre [2], [3], [4]. Not surprisingly, therefore, there is considerable interest in the enzymatic synthesis and degradation of mannosides. In the context of the glycoside hydrolase (GH) catalyzed α−mannosidase hydrolysis, several of the >100 GH sequence-based families (www.cazy.org [5]; reviewed in [6], [7], [8], [9]) contain enzymes with putative α−mannosidase activity. These families include GH38 configuration-retaining α−mannosidases and family GH47 inverting mannosidases, that together are the most studied of the known α−mannosidases, as well as enzymes in GH76 and GH92; the latter recently studied in the context of 3-D structure, specificity and catalysis [10].

**Figure 1:**
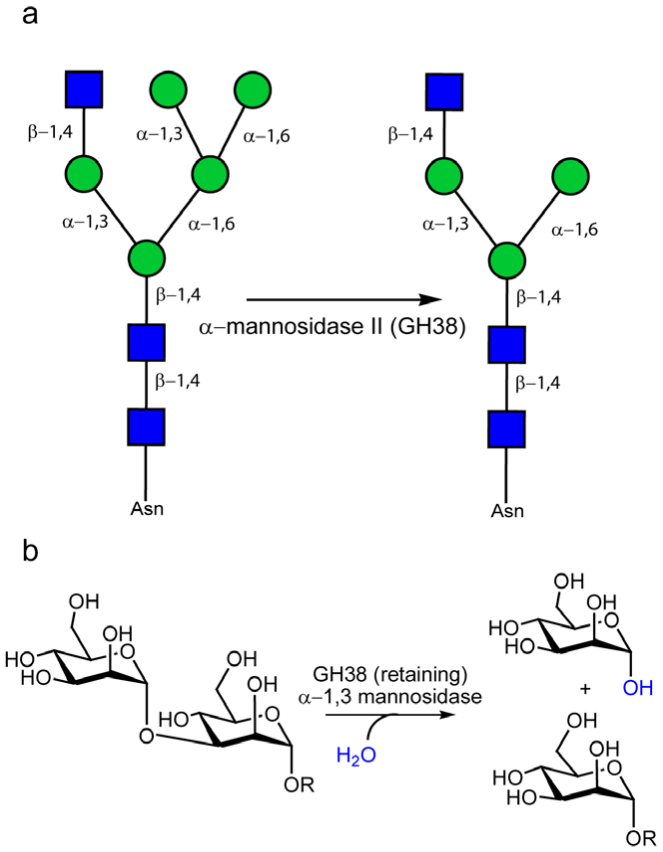
Catalytic activity of GH38 α−mannosidases. (**A**) Golgi α−mannosidase II is responsible for the hydrolysis of both α−1,3 and α−1–6 mannosides during the diversification of hybrid N glycans (GlcNAcMan_5_GlcNAc_2_ becoming GlcNAcMan_3_GlcNAc_2_). (B) The catalytic action of a retaining α−mannosidase, here exemplified for the α−1,3 mannosidase activity of GH38 enzymes; catalysis occurs with net retention of anomeric configuration.

Of particular relevance to the study described here, are the GH38 α−mannosidases. Based on sequence similarity, these enzymes are approximately 1000 residues in length and have been found across the Archaea (19 ORFs, as of 18-Jan-2010), Bacteria (295 ORFs) and Eukaryote (118 ORFs) domains of life. The higher eukaryotic GH38 enzymes are involved in the processing the high-mannose and hybrid *N*-glycans, **Figure 1**. During cancer metastasis, the degree of branching in *N*-linked carbohydrate structures has been correlated with malignancy and disease progression through disruption of normal intracellular interactions and effectively concealing cancerous cells from immune detection [11]. Furthermore, mouse knock-outs show that mannosidase II deficient animals suffer from lupus-like auto immune diseases [12]. Characterisation of the mechanisms responsible for the synthesis, breakdown and recognition of oligosaccharides in *N*-linked glycosylation has therefore inspired considerable interest in the structural enzymology of GH38 enzymes.

At the 3-D level, the *Drosophila* α−mannosidase II [13] is the most extensively studied GH38 enzyme. This Golgi localised enzyme has dual α−1,3 and α−1,6 mannosidase activity and is implicated in the maturation/diversification of “hybrid” *N-*glycans prior to their augmentation into complex glycan structures, **Figure 1**. The initial *Drosophila* α−mannosidase II structure was solved in 2001 [14] and this has been followed by several 3-D analyses designed primarily to probe sub-site specificity [15] and the chemical [16] and conformational [17] aspects of mannosidase catalysis as well as α−mannosidase inhibition [18], [19], [20], [21], [22], [23], [24], [25], [26]. α−mannosidase II is considered a potential anti-cancer target, not least because its action in hybrid *N*-glycan modification is required prior to metastatic changes in *N*-glycans; such as those involving GlcNAc Transferase V [27], [28], [29], [30]. Indeed, the anti-cancer indolizidine alkaloid, swainsonine, is a potent inhibitor of α−mannosidase II and has been shown to reduced metastasis and improved clinical outcomes when used in clinical trials for treatment of colon, breast, and skin cancers [11]. The GH38 bovine lysosomal α−mannosidase (bLAM) has also been subjected to biochemical and structural analysis [31]. Despite the complexities arising from the proteolytic cleavage of the bovine enzyme into five fragments, the 3-D structure of bLAM confirms a similar overall domain structure and catalytic center for the mammalian enzyme. Yet, despite this wealth of structural information, little is known of the bacterial GH38 enzymes, many of which come from human symbionts and pathogens; although the link between pathogen carbohydrate catabolism and pathogenesis is well documented (for example refs. [32], [33]).

The CAZy website (www.cazy.org) reveals that many bacterial species encode GH38 enzymes. At the time of submission very few of the ∼300 open reading frames encoding putative GH38 α−mannosidases from bacteria have been characterised in any way. One exception, however, is the *Escherichia coli* α−mannosidase MngB, which converts 2-*O*-(6-phospho-α-mannosyl)-D-glycerate to mannose-6-phosphate and glycerate in the pathway which enables use of mannosyl-D-glycerate as a sole carbon source [34]. Other bacterial GH38 representatives are from a diversity of genera including *Bacteroides* (4), *Clostridia* (13), *Listeria* (28 with 4 or 5 GH38 entries per *Listeria* species), and *Mycobacteria* (20). However, with 32 species represented out of 37 possible, the genus *Streptococcus* constitutes the highest proportion of bacterial GH38s, including many human pathogens such as *S. pyogenes* (Group A *S* *treptococcus* or GAS). Group A streptococci are the pathogenic bacteria responsible for many acute human infections in the respiratory tract and skin including pharyngitis, impetigo, rheumatic fever, and acute glomerulonephritis [35]. Alarmingly, since the 1980s *S. pyogenes* has been identified to be globally responsible for a class of emerging, life threatening, invasive infections including the “flesh-eating” disease, necrotizing fasciitis, septicemia, and the excretion of the pyrogenic exotoxin-associated toxic shock syndrome [35]. Treatment of these invasive diseases, even with broad spectra antibiotics, is not always effective, with patient mortality exceeding 80% in cases of toxic shock [36]. The paucity of information on the α−mannosidases from these pathogenic bacteria, led us to study the *S. pyogenes* SpGH38 enzyme Spy1604. The SpGH38 gene is located on an operon that contains (in addition to two sugar transporters, transcriptional regulators and two-component putative histidine kinase) two other glycoside hydrolase genes. These are the GH84 Spy1600 enzyme, which is known to be a hexosaminidase that is able to cleave β−linked *N*-acetylglucosaminyl moieties from diverse substrates [37] and a GH1 “putative β−glucosidase” (a family that includes varied β−D glycoside hydrolases including glucosidases, mannosidases, galactosidases and glucuronidases).

Here we report, what we believe to be, the first structure of a bacterial α−mannosidase from the human pathogen *S. pyogenes* bound to the therapeutically important inhibitor swainsonine. Comparison of SpGH38 with *Drosophila* Golgi α−mannosidase II suggests a conserved domain architecture and catalytic centre in which diversification in the “leaving group” subsites accounts for the subtly different specificity. We show that SpGH38 is specific for α−1,3 linkages, with high activity on an α−1,3 disaccharide but no appreciable activity on α−1,6 linked substrate. SpGH38 is also able to hydrolyse (Man)_5_(GlcNAc)_2_ *N*-glycans to (Man)_3_(GlcNAc)_2_ consistent with an α−1,3 mannosidase activity, **Figure 1b**. Together with the operon organisation, the data imply that SpGH38 is a component of a host *N*- or possibly *O*-glycan degradation system.

## Results and Discussion

### SpGH38 Sequence Analysis

*Streptococcus pyogenes* M1 GAS SF370 ORF Spy1604 encodes a putative GH38 α-mannosidase. The gene encoding the full length enzyme (901 amino-acids) was cloned and subsequently over-expressed using a York ligation-independent cloning strategy (Ysbl-LIC) [38], [39]. Protein was produced at high levels in *E. coli* and purified using metal-ion affinity and gel filtration chromatography (see Materials and Methods).

SpGH38 is distantly related to various mammalian and insect α−manosidases including homologues from *Bos taurus* (2 ORFs), *Drosophila* (8), and *Homo sapiens* (5), as well as a variety of plant enzymes including α−mannosidases from *Arabidopsis* (4) and *Oryza sativa* (4) where numbers in brackets represent the number of family GH38 representatives from each species. These higher eukaryotic GH38s are quite divergent, reflected in, for example 28 and 24% sequence similarity with bLAM and dGMII respectively whilst within a “compartment” (such as human vs. *Drosophila* golgi enzymes) the identity is typically far greater (>40%). Within the *Streptococcal* species, sequence analyses reveals known GH38 homologs in group A *S. pyogenes*, group C *Streptococcus* (YP_002997299), group D *Streptococcus* (ZP_03980341), and also non-hemolytic *Streptococcus* (YP_002349517). Within *S. pyogenes* strains, GH38 ORFs are observed in *S. pyogenes* serotypes (M49 591, NZ131, MGAS9429, Manfredo, MGAS10394, MGAS8232, MGAS10270, MGAS6180, MGAS10750, MGAS315; see www.cazy.org.

### Catalytic Activity of SpGH38

**Figure 2:**
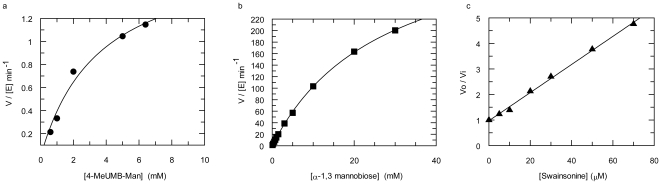
Catalytic activity of SpGH38 α−mannosidase and inhibition by swainsonine. (**A**) Activity on 4-methylumbelliferyl α−D mannoside (4-MeUMB) and (**B**) α−1,3 mannobiose (see text for details) Substrate insolubility under the conditions used precluded higher [S] values. (**C**) The *K*_i_ for swainsonine was determined using α−1,3 mannobiose as substrate with [S] < < K_m_ and [I] straddling the *K*_i_. V_0_ and V_i_ are the rates of the reaction in the absence and presence of inhibitor, respectively. The *K*_i_ for a competitive inhibitor is derived from the gradient of 1/*K*_i_ (see Methods ); here 18±0.5 µM.

Recombinant SpGH38 was tested for α−mannosidase activity initially on a range of aryl α−mannosides (4-nitrophenyl α−D mannoside, 2,4 dinitrophenyl α−D mannoside and 4-methylumbelliferyl α−D mannoside). Activity was detectable, but poor, on both the 2-nitrophenyl and 2,4 dinitrophenyl derivatives and this prevented calculation of individual *k*_cat_ and *K*_M_ values for either substrate. The 2,4 dinitrophenyl substrate alone allowed calculation of an approximate *k*_cat_/*K*_M_ of ∼0.6 min^−1^mM^−1^. Activity on 4-MeUMB-Man was greater, permitting full Michaelis-Menten kinetics and allowing description of the SpGH38 as an α−mannosidase with a *k*_cat_ of 1.8±0.24 min^−1^ and *K*_M_ of 3.6±1.0 mM, **Figure 2**. SpGH38 activity was subsequently measured on a range of commercial α−mannobioside disaccharides (1,2/1,3/1,4 and 1,6 linked). The enzyme showed significant activity only on the α−1,3 linked disaccharide substrate yielding *k*_cat_ of 384±6.2 min^−1^ and *K*_M_ of 27±0.75 mM, **Figure 2b** **.** The data show that SpGH38 acts as an effective α−1,3 mannosidase with a *k*_cat_/*K*_m_ of 14.2 min^−1^mM^−1^ on the disaccharide.

**Figure 3:**
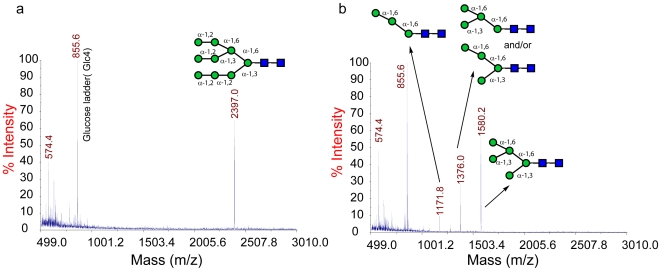
SpGH38-catalysed hydrolysis of Man_9_(GlcNAc)_2_ glycans. (**A**) Action of SpGH38, alone, on Man_9_(GlcNAc)_2_. The glycan remains unmodified. (**B**) Action of SpGH38 in combination with a specific α−1,2 mannosidase the *Bacteroides thetaiotaomicron* Bt3990. Following α−1,2 mannoside removal (which has previously been shown to be specific, see Supplemental Figure 1 in [10], SpGH38 is able to further degrade the unmasked glycans, with the action pattern most indicative of α−1,3 mannosidase activity. An α−1,3 mannosidase activity for SpGH38 is further supported by the specificity of the enzyme for the disaccharide α−1,3 mannobiose (see text).

In order to test the activity and specificity of the enzyme on human *N*-glycans an unmodified human Man_9_GlcNAc_2_ glycan was incubated with SpGH38. No activity on the intact glycan was observed, **Figure 3a**, typical for many α−mannosidases whose lack of α−1,2 mannosidase activity prevents access to these masked substrates. The Man_9_GlcNAc_2_ glycan was therefore co-incubated with a specific α−1,2 mannosidase, the *Bacteroides thetaiotaomicron* Bt3990 enzyme (YP_210385) [10], to generate an “unmasked” Man_5_GlcNAc_2_ glycan (m/z 1580.2, **Figure 3b**). This was indeed a substrate for SpGH38 which was able to degrade it further to Man_4_GlcNAc_2_ (m/z 1376.0) and Man_3_GlcNAc_2_ (m/z 1171.8) **Figure 3b**. Although one cannot formally exclude a small amount of α−1,6 activity, in light both of the previous observations on mannobioside specificity and the absence of any significant amount of further degradation product, the most likely interpretation of the mass spectrometry data is that SpGH38 is able to sequentially remove the two α−1,3 linked mannobiosyl moieties from Man_5_GlcNAc_2_ to yield a glycan in which the α−1,6 linked mannosides remain, **Figure 3b**. This α−1,3 specificity is also in agreement with the active centre topography, described below in light of the 3-D structure of the SpGH38 enzyme and a comparison with the dual α−1,3/α−1,6 mannosidase in CAZY family GH38 – the *Drosophila* α−mannosidase II.

### SpGH38 Structure Determination

The three-dimensional structure of SpGH38 was determined by the multiple anomalous dispersion phasing method using a selenomethionine-derivative form of the protein and the native structure refined to 1.90 Å resolution, **Table 1**. Subsequently, an inhibited form of SpGH38 in complex with swainsonine was determined at 2.6 Å resolution (discussed below). The selenomethionine-derived and native forms of SpGH38 each crystallize in different space groups, tetragonal *P*4_3_2_1_2 and monoclinic *P*2_1_, respectively, each with two protein molecules in the asymmetric unit. In each crystal form, the two SpGH38 molecules within the asymmetric unit inter-digitate to form a dimer. These dimers are essentially identical between crystal forms, overlapping with a root mean squared deviation of 0.4 Å on C-alpha atoms. Furthermore, molecular weight estimation for SpGH38 by size exclusion chromatography indicated a molecular mass of 250 kDa (data not shown). This suggests that SpGH38, with a monomer molecular weight of 102,751, behaves as an elongated dimer in solution. Of the GH38 structures of known function, the Bos Taurus bLAM [31], is also believed to be a dimer, whereas the *Drosophila* dGMII is believed to be monomeric [14].

**Table 1:** Data collection and refinement statistics for the *Streptococcus pyogenes* GH38 α−mannosidase.

Data collection	Se “Peak”	Native	Swainsonine
Resolution range (Å)	50–3.00 (3.11–3.00)	50–1.90 (1.97–1.90)	50–2.60 (2.69–2.60)
Space group	P4_3_2_1_2	P2_1_	P4_3_2_1_2
Unit cell dimensions			
a, b, c (Å)	180.8, 180.8, 194.7	92.6, 88.5, 134.7	178.7, 178.7, 198.2
α, β, γ (°)	90, 90, 90	90, 109.0, 90	90, 90, 90
Completeness (%)	100 (100)	99.4 (99.1)	99.8 (100)
R_merge_	0.088 (0.24)	0.052 (0.45)	0.053 (0.39)
Redundancy	16.3 (16.6)	3.3 (3.0)	6.8 (7.0)
I/σ(I)	20.0 (11.2)	14.4 (2.0)	25.7 (4.1)
Refinement	n/a		
Resolution range (Å)		50–1.90 (1.97–1.90)	50–2.60 (2.69–2.60)
Unique reflections		152396	93181
R_work_/R_free_ (%)		17.8/ 20.5	18.7/22.3
Rmsd bond lengths (Å)		0.009	0.009
Rmsd bond angles (°)		1.1	1.1
Number of atoms			
Protein		14433	14790
Ligand/ion		0/2	24/2
Solvent		995	417
Average B factors (Å^2^)			
Protein		16	30
Ligand/ion		—/29	50/72
Solvent		21	34
Rmsd B bonded-atoms (main-chain/side-chain/ligand)		0.44/1.4/—	0.37/1.0/1.2
Ramachandran plot (%)			
Most favored		96.1	96.3
Allowed		3.3	3.4
Outliers		0.6	0.3
PDB Code		2WYH	2WYI

**Figure 4:**
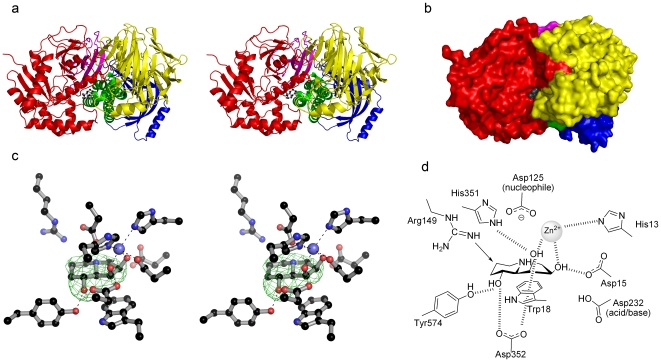
3-D structure of SpGH38 and its swainsonine complex. (**A**) 3-D topology cartoon (divergent stereo) colored according to domains with swainsonine in ball-and-stick. N-term (red: 1–294) 3-α (green: 295–392), β-1 (blue: 393–514,806–825), β−2 (yellow: 522–805) and β−3 (cyan: 825–901) (**B**) Surface representation of SpGH38 colored as for part (A). (**C**) Active centre and electron density for the Swainsonine/Zn^2+^ complex of SpGH38 (divergent stereo). The map shown is the unbiased F_obs_-F_calc_ synthesis, contoured at 2.5 σ, calculated with model phases prior to the incorporation of Swainsonine/Zn^2+^ in any refinement. (**D**) schematic diagram of the interactions of swainsonine (shown in panel C) with H-bonds >3.0 Å shown as dashed lines and residue numbers for the SpGH38 indicated. Arg149 makes a close contact to a swainsonine carbon (indicated with an arrow) of 2.9 Å (spatially equivalent to an H-bond to mannose O6 of the true substrate).

Although exact delineation of domains is subjective, the SpGH38 structure can be considered as five domains: an N-terminal α/β-domain (residues 1-294), a three-helix bundle (295–392) and three predominantly β -sheet domains (393–515/806–824, 522–805, 825–901), **Figure 4**). The N-terminal α/β-domain, three-helix bundle, and the β−2 and β−3 domains form the “core” of SpGH38 with all of these domains contributing to the active center and substrate binding surface. The β−1 domain bows outward from the protein core, is involved in dimer interactions whilst also forming a lid “above” and somewhat into the active centre of its dimer mate. Broadly speaking SpGH38 resembles an elongated ellipse that is convex along the surface where β−2 and β−3 domains interact, and concaved on the three-helical bundle exposed surface.

The α/β-domain features a distorted β-barrel core composed of primarily parallel strands, surrounded by eight helices of varying length and an appreciable amount of coil. A metal ion, known to be catalytic and presumed to be zinc (see below) is coordinated by three residues derived from the coil elements: His13, Asp15 and Asp125, together with a single residue, His351, from a loop of the helical-bundle domain (**Figure 4a, b**). The three-helical bundle runs across the narrow axis of the concaved surface of SpGH38, serving as a central structural feature that makes many interactions with each of the other domains. The β−1 domain consists of seven anti-parallel strands which form a twisted β-barrel in which three of the strands along one side of the barrel extend beyond the edge of the barrel, twisting away from the core. An α-helix connects the two sides of the β1-barrel at this distal opening. Due to a lack of clear electron density, a gap exists in the model between residues 515–521 which join the β−1 and β−2 domains. Central to the β−2 domain is a 17-stranded, twisted β-super-sandwich, punctuated by an α-helix that joins strands 7 and 8, and terminating at another helix before forming a strand that contributes to the β−1 domain. The C-terminal β−3 domain contains four twisted anti-parallel β-strands and three extended coils resembling a distorted β-sandwich. Furthermore, axial to the β−1 domain and bordered by the three-helix and β−2 domains, a narrowing, cone-shaped channel through SpGH38 is established. This structural feature is of unknown function but is conserved in dGMII [14] and bLAM, albeit narrower in the latter case.

### Comparison of SpGH38 to Known Structures

Secondary structure matching of full length SpGH38 using SSM [40] not surprisingly reveals the GH38 *Drosophila* α−mannosidase II as the top “hit” (Z score 9.8 with 664 residues aligning, r.m.s.d 2.6 Å). Due to the bLAM structure being defined as five separate chains in the PDB deposited coordinates it was not highlighted from an initial SSM search. However, pair-wise alignment of SpGH38 and bLAM is comparable to that observed for dGMII (Z score 8.6 with 686 residues aligning, r.m.s.d 2.8 Å. Beyond the GH38 family, very distant similarity is observed the *Bacteroides thetaiotaomicron* α-glucosidase (GH97), and this is largely due to similarity with the β−2 and β−3 domains of SpGH38.

Overall, the individual domains and architecture of SpGH38 are similar to both dGMII and bLAM, albeit different in spatial arrangement and intra-domain contacts, overlapping full length secondary structures with mean square deviations of 2.6 and 2.8 Å, respectively. However, the structural divergence was significant enough to prevent either being used as a suitable molecular replacement model for SpGH38, even when shorter, more conserved, features were used. Crystallization and diffraction data collection have also been reported for two other GH38 family members: the cytosolic α-mannosidase TM1851 from *Thermotoga maritima* and the *Saccharomyces cerevisiae* α-mannosidase Ams1 [41], [42]. However, molecular replacement using either dGMII or bLAM failed to result in a solution for either case.

As discussed, SpGH38 appears to exist as an elongated dimer. The SpGH38 dimer forms through an inter-digitation of the β−3 domain into the opening formed between α/β and β−2 domains of an adjacent molecule. A series of van der Waals' and hydrogen bonding interactions stabilize this intra-molecular connection involving hydrophobic residues Y574, V629, W764, Y766, F767 from the α/β and β−2 domains with F429, F433, P434, Y438, F474, Y476, L478, P479, F483, P486, and F488 of the β−3 domain which would be surface exposed in a monomeric state. This insertion of the β−3 domain effectively reduces the area of the putative Man_5_GlcNAc binding site in an adjacent molecule and introduces Y476, F483, R484, and Q431 which could putatively stabilize GlcNAc and Man substituents thereby anchoring a high mannose substrate. This inter-digitation between adjacent crystallographic dimers could also suggest a mechanism for restricting substrate access to the active centre, thereby providing added substrate specificity.

The putative high mannose anchoring site in bLAM is similar to SpGH38 in that it extends longer and wider compared to dGMII. Furthermore, although bLAM exists as a monomer in the crystal state, it has been characterized to be a dimer in solution. Through EM and crystal packing bLAM was hypothesized to form two possible dimers; one in which contacts are made between regions equivalent to the β−2 and β−3 domains in SpGH38, and another in which contacts are made between equivalents to the α/β-domain, the latter of which was more favored. The dimerization observed for SpGH38 introduces an alternative possibility for bLAM oligomerization.

### Active Center and Swainsonine Complex

**Figure 5:**
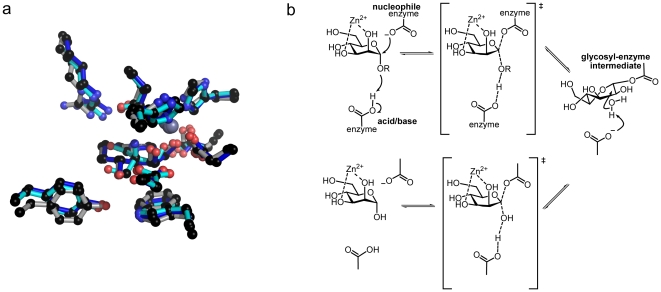
Conservation of GH38 reaction mechanism. (**A**) Conserved active-centre constellation (here -1 subsite only) between the SpGH38 (grey), bovine bLAM (cyan) and the *Drosophila* GH38 α−mannosidase (blue). (**B**). GH38 α−mannosidases are known to act with net retention of anomeric configuration; a mechanism in which a glycosyl-enzyme intermediate is flanked by oxocarbenium-ion like transition-states. The intermediate has been trapped by the Withers and Rose groups [17] and shown to bind in a ^1^S_5_ skew-boat conformation which in the absence of evidence to the contrary might imply a transition-state close to a B_2,5_. Pseudo-Michaelis complexes published on the *Drosophila* α−mannosidase II show the −1 sugar in a ^4^C_1_ chair conformation but these have been obtained on a nucleophile-alanine variant so their conformational relevance to catalysis is unclear [15].

The natural product swainsonine is known to be a versatile glycosidase inhibitor having previously been used to study the active center of the *Drosophila* enzyme [14]. On SpGH38 it displays a *K*_i_ value of 18 µM (**Figure 2c**) using the linked assay with α−1,3 mannobiose as substrate. A complex was obtained of swainsonine with SpGH38 and data collected to 2.6 Å resolution. The mean B value for the ligand, 52 Å^2^, equates to that of the dataset as a whole (51 Å^2^) but that it is higher than its surroundings hints at an occupancy <1 for both swainsonine and the Zn^2+^ ion; the unbiased F_obs_-F_calc_ density is, however, unambiguous, Figure 4c. The active center of SpGH38 is located in a cleft at the bottom of a broad surface channel between the α/β− and β2-domains and bordered by a short helical linker that joins the second and third helices of the three-helical bundle domain. In apo-SpGH38 the zinc ion is coordinated in T_5_-square-based pyrimidal geometry by the OD1 moieties of D15 and D125, the NE2 nitrogens of H13 and H351, and a water molecule analogous to that characterized for dGMII [14], **Figure4c, d**; **Figure 5a**. In the swainsonine complex the position of the water molecule is occupied by the O2 hydroxyl oxygen of swainsonine. An additional contact is established between the O3 hydroxyl oxygen of the five-membered ring and zinc, which is now in an overall T_6_-octahedral coordination arrangement. The O1 hydroxyl oxygen of swainsonine is stabilized through electrostatic interactions with side chain oxygens of Y574 and D352 and further contributions from D15 and D352 stabilize the O3 and O2 hydroxyl oxygens of swainsonine in the active centre, respectively (schematic diagram in **Figure 4d**). These active centre interactions with hydroxyl oxygens 1–3 of swainsonine correspond to analogous interactions between dGMII and hydroxyl groups at positions 4, 3, and 2 of α−mannose in the -1 subsite [17] (subsite nomenclature in Ref. [43]), which helps to explain why swainsonine represents such a useful inhibitor of α-mannosidase II.

In addition to the favorable electrostatic interactions discussed previously, the position of F127 is conserved across α-mannosidase II active centers. Additionally, swainsonine is stabilized through stacking interactions of W18 with the five-membered ring, suggesting that the two aromatic residues contribute to positioning mannose into the correct orientation in the −1 subsite. Other conserved active centre residues, D125 and D232 are in equivalent positions to the catalytic nucleophile and general acid/base, respectively, of dGMII. As with the *Drosophila* enzyme, it is likely that R149 will encourage D125 to be ionized (as required for a nucleophile). The hydrogen-bond from Y192 to D125 may also stabilize the ionized form of D125. D232 will likely be neutral due to negative charge repulsion from D125, as expected for the acid/base.

The structure of SpGH38 is consistent with previous proposals (notably [17]) concerning the chemical basis for catalysis with net retention of anomeric configuration in this family. Thus one can support a mechanism, **Figure 5b**, in which protonic assistance to leaving group departure is given by D232 with electrophilic migration of the anomeric carbon to form the covalent glycosyl-enzyme intermediate. It seems likely that Zn^2+^ plays a role in aiding distortion of the sugar towards the transition-state (as also discussed for the Ca^2+^ in GH92 [10] and GH47 [44] α−mannosidases). The covalent intermediate species was trapped and observed on the *Drosophila* enzyme and shown to bind in a ^1^S_5_ (skew-boat) conformation [17]. Based on the stereoelectronic requirements for an incipient oxocarbenium-ion, the ^1^S_5_ intermediate was thus interpreted as implying catalysis “around” the B_2,5_ boat conformation. Such a proposal is consistent with diverse work on β−mannosidases in which β-linked substrate complexes have been observed in the ^1^S_5_ conformation (for example [45], [46], [47] and transition-state mimics ion B_2,5_ (Tailford et al. 2008), although such proposals are objected to by one group [48]. Catalysis around this area of the conformational sphere has also been proposed, recently, for inverting GH92 α−mannosidases [10]. The conformation of the Michaelis complex in GH38 enzymes is more difficult to address since the known 3-D structures of the *Drosophila* enzyme are observed with a nucleophile-Ala variant with the −1 subsite sugar (in ^4^C_1_ chair conformation) occupying a position that would not be possible on a wild-type enzyme [15]. Despite these caveats, the +1 subsite is well-mapped in these complexes and lies “below” the plane of the −1 sugar in a position consistent with an axial bond to the leaving group.

### Basis for Apparent Substrate Specificity in SpGH38

**Figure 6:**
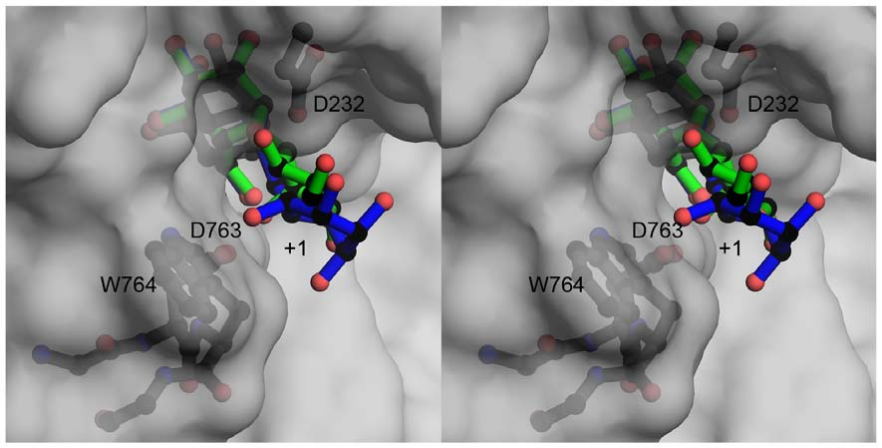
Substrate specificity in SpGH38. An overlap of the *Drosophila* GH38 α−mannosidase II complexes [15] with α−1,3 (green bonds) and α−1,6 linked ligands (blue bonds) with the SpGH38 structure (grey surface) focussing on the +1 subsite (the -1 subsites are essentially identical, Fig. 5A). Features which may contribute to 1,3 specificity include the position of W764, the interactions afforded by D763 and the tightness of the “sphincter” formed by D763 and the catalytic acid/base D232. The figure is shown in divergent (“wall-eyed”) stereo.

In the −1 subsites, the *Drosophila* and SpGH38 enzymes are extremely similar, reflecting the common recognition and catalysis of α−mannosides, discussed above. The *Drosophila* GH38 α−mannosidase II is a dual specificity α−1,3/α−1,6 mannosidase whereas, on disaccharide substrates and *N*-glycan models SpGH38 appears to display α−1,3 mannosidase activity. The extended binding sites of the *Drosophila* enzyme are indeed formed by elements of structure not present in the less-decorated Streptococcal enzyme. Ala189 at the end of a core β−strand makes a structural divergence between the two enzymes in which the *Drosophila* enzyme (Pro265) embarks on a long insertion to Arg314 compared to a much shorter loop region to Glu204 in the Streptococcal enzyme. This extended loop region, together with variations elsewhere (notably 408–413) and a long N-terminal extension provide for considerably more developed leaving group subsites, and likely more sophisticated leaving group recognition, in the *Drosophila* enzyme compared to SpGH38. Furthermore, although the loops from (SpGH38) 757–773 (equating to approximately 859–883 in *Drosophila* α−mannosidase II) are of similar length, they vary markedly in orientation with elements of the SpGH38 sequence, notably W764 clashing into the +1/+2 subsites of the *Drosophila* 1,6 linked substrate complex (PDB pdb code 3bvw) – but making far less extensive clashes with the *Drosophila* 1,3 linked substrate complex (pdb code 3bvv), **Figure 6** **.** Within this region lies D763 of the SpGH38 whose, side-chain interacts would interact with the 2-OH of the +1 subsite mannoside, if one compares with the α−1,3 linked complex of the *Drosophila* enzyme. In the bovine and *Drosophila* enzymes the equivalent interaction is achieved via the main-chain carbonyl group of R876 (*Drosophila* numbering) with O2 (dist ∼2.7 Å). In addition, the substrate binding cleft is more tightly constricted in the SpGH38, at this position, with just 6.2 Å between the catalytic acid/base (E and the putative O2 interacting D763 carboxylate oxygen compared to 7.5 Å for the comparative constriction in the *Drosophila* enzyme; a feature used in this latter case to accommodate the different α−1,3 and α−1,6 linked substrates. It is thus possible that the carboxylate of D763 could also make specific, productive interactions with α−1,3 linked mannoside (O2) compared to an α−1,6 linked substrate and that binding of the latter is hindered both by a tighter “collar” and the location of W764, but such speculation requires the analysis of complexes of the Streptococcal enzyme. As yet, we have not been able to obtain “leaving group”-containing complexes. One possible reason for this may be the insertion of the β−1 domain into the active centre of the dimer mate in the crystal forms observed here. In particular, Arg484 from this loop stacks with W764 discussed above, thus reducing accessibility to the +1 subsite in-crystal. Given the high *K*_M_ (27 mM) for the disaccharide, it would seem likely that a more extended substrate is favored *in vivo* but it is difficult to speculate more on the exact nature of these addition subsites at this point.

### Summary

We have shown that The *S. pyogenes* ORF *spy1604* encodes an α−1,3 mannosidase that is active on disaccharides, some aryl glycosides and can also effectively deglycosylate human *N*-glycans *in vitro*. This, coupled to the presence of a gene encoding an *N*-acetyl glucosaminidase (Spy1600) on the operon (an enzyme known to break β−linked *N*-acetylglucosaminides [37]) would suggest a role, *in vivo*, in the utilization of human glycan-derived carbohydrates as a nutrient source. It is known, for example, that other Streptococcal species can utilize *N*-glycans as their carbon sources; *Streptococcus oralis* grown using *N*-glycosylated ribonuclease B as the sole carbohydrate source produces α−1,3/α−1,6 and β−1,4 mannosidase activities to harness the *N*-glycans [49]. What is less clear is the cellular location of these enzymes. The absence of a secretion signal on SpGH38 and the GH84 hexosaminidase suggests, in the absence of evidence to the contrary, an intracellular location (although one cannot rule-out non-canonical sortase-based extracellular display, as recently shown for *Streptococcus pneumoniae* O-glycan degradation [50]). *Streptococcus pyogenes* does produce at least one secreted *N*-glycan deglycosylation enzyme. The most notably of these is EndoS, a family GH18 enzyme that is a key virulence factor in the organism [33], not least because of its ability to deglycosylate host immunoglobulins [51] leading to immune impairment and bacterial persistence. One possible role for the GH38 α−mannosidase and GH84 β−GlcNAcase enzymes might therefore be in the subsequent intracellular utilization of *N-*glycan oligosaccharides, derived from the extracellular action of EndoS or related enzymes.

## Materials and Methods

### Gene Cloning

The sequence of full length *S. pyogenes* α-mannosidase II, coding for SpGH38 residues 1-901, was amplified by PCR from genomic DNA using KOD Hot Start DNA polymerase (Novagen) and complementary gene-specific primers with additional 5′ sequences to facilitate ligation-independent cloning (LIC) as follows (with the LIC overhangs underlined:

5′-CCAGGGACCAGCAATGGCAACTAAAAAAGTACATATTATTTCACACAGTC-3′,

5′-GAGGAGAAGGCGCGTTATTGTTTCTTCCAAGCTAGAGTTAAAATTTCC-3′

The DNA product was then treated with T4 DNA polymerase in the presence of dATP to generate single-stranded 5′ overhangs. Subsequent treatment with *Bse*RI resulted in complementary overhangs necessary for incorporation into the modified *Escherichia coli* expression vector pET28a, pET-YSBLIC [38]. LIC incorporation was facilitated by treatment with T4 DNA polymerase in the presence of dTTP to generate pSpGH38. The pSpGH38 expression construct under control of a T7 promoter contains SpGH38 fused to a 3C protease cleavable N-terminal His_6_-tag (MGSSHHHHHHSSG**LEVLFQGP**A-*Sp*GH38), where the rhinoviral 3C protease recognition site is shown in bold. The SpGH38 sequence was analyzed with help of the CAZy database [5] and BlastP [52]. Sequence alignments were conducted with ClustalW [53].

### Gene Expression and Protein Purification

*E. coli* BL21:DE3 cells harboring pSpGH38 were cultured in Luria broth supplemented with 50 µg mL^−1^ kanamycin at 37°C to mid-exponential phase (*A*_600_ ∼0.6). The temperature was reduced to 16°C for 1 h at which time recombinant SpGH38 was induced by the addition of 0.2 mM isopropyl 1-β-D-thiogalactopyranoside and incubated for sixteen additional hours. Pelleted cells were resuspended in 50 mM NaH_2_PO_4_, 300 mM NaCl, pH 7.5 (buffer A), disrupted through sonication, and the clarified supernatant loaded onto a nickel-immobilized HiTrap Chelating™ 5 mL column pre-equilibrated with buffer A on an ÄKTA Explorer (Amersham Biosciences) FPLC. The lysate-loaded column was washed extensively with buffer A supplemented with 20 mM imidazole and 50 mM imidazole, in step-wise fashion, and SpGH38 eluted with 500 mM imidazole. Eluted fractions were passed to a Sephacryl S-200 gel-filtration column pre-equilibrated with 25 mM Tris, 150 mM NaCl, pH 7.5 for further purification. Fractions containing the α-mannosidase were identified by SDS-PAGE, pooled and concentrated using a 10 kDa MWCO Vivaspin 20 centrifugal concentrator. Swainsonine-complexed crystals were obtained from protein cleaved with 0.1 mg mL^−1^ 3C protease at 4°C overnight, that was subsequently dialyses into 25 mM Tris (pH 7.5) prior to concentration.

### Aryl Mannoside Kinetics and Determination of Inhibitor *K*_i_ Values

The activity of SpGH38 against 4-nitrophenyl-α-D-mannopyranoside (PNP-Man) or 2,4-dinotrophenyl-α-D-mannopyranoside (DNP-Man) were determined at 37°C in 50 mM NaH_2_PO_4_, pH 6.8, containing 1 mg mL^−1^ bovine serum albumin and substrate concentrations ranging up to 1.6 and 4.0 mM, respectively. 4-Methylumbelliferyl α-D-mannopyranoside assyas were conducted at 37°C in 100 mM MOPS, pH 7.0, containing 0.1 mM ZnSO_4_ and 1 mg mL^−1^ bovine serum albumin, respectively. The total reaction volume was 500 µl. Methylumbelliferone (MU; ε_365_  = 2.99×10^8^ M^−1^ cm^−1^ at pH 7.0) derived from MU-Man hydrolysis was measured continuously by a fluorimeter with an excitation wavelength of 365 nm and an emission wavelength of 440 nm. Swainsonine (obtained from GlycoFineChem, New Zealand) inhibition assays were carried out using the α−1,3 mannobiose substrate with mannose detection as described below. The inhibition reactions were carried out with α−1,3-mannobiose as substrate and a range of inhibitor concentrations that spanned the *K*_i_, which was calculated using the following equation: 
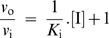
where ν_0_ and ν_i_ are the rates of the reaction in the absence and presence of inhibitor, respectively. Under conditions where [S] < < *K*_M_, the fractional decrease in rate thus yields the *K*_i_ for a competitive inhibitor. A graph plotting *v*_0_/*v*_i_ on the y-axis against the concentration of inhibitor on x-axis will give a gradient of 1/*K*_i_, and an intercept of 1.

### D-Mannose Detection Assay

SpGH38 specificity for α−1,2-, 1,3-, 1,4- or 1,6-linked mannobiose was determined using a four enzyme coupled assay based on the Megazyme International kit for D-mannose detection, deploying ATP and NADP^+^. Reactions were conducted at 37°C in 100 mM MOPS (pH 7.0), containing 0.1 mM ZnSO_4_, 1 mg mL^−1^ BSA, and 0.2 µM SpGH38. Briefly, SpGH38 liberated mannose was phosphorylated to mannose-6-phosphate by hexokinase (HK) which was subsequently converted to fructose-6-phosphate through the action of phosphomannose isomerase (PMI). Fructose-6-phosphate was then isomerized to glucose-6-phosphate by phosphoglucose isomerase (PGI) and finally, oxidized to gluconate-6-phosphate by glucose-6-phosphate dehydrogenase (G6P-DH). The G6P-ΔH NADP^+^ cofactor is concurrently reduced to NADPH, which was monitored at 340 nm using an extinction coefficient of 6223 (M^−1^·cm^−1^). The enzymes were individually obtained from Sigma Chem. Co. and the concentrations (5U final in each case) were selected such that disaccharide cleavage was the rate limiting step in the reaction.

### Enzyme Activity on High Mannose *N*-glycans, Mass Spectrometric Analysis of the Reaction Products

High mannose *N*-glycans (2–2.5 µg) were incubated with 2 µM SpGH38 at 37°C overnight in 50 mM MOPS, pH 7.0, containing 0.1 mM ZnSO_4_ and 1 mg mL^−1^ bovine serum albumin. The reaction products were lyophilized overnight, and then submitted to MALDI-TOF for analysis after permethylation. The procedure for preparing Man_5_GlcNAc_2_ is as follows: 2.5 µg of Man_9_GlcNAc_2_ (obtained from Dextra Laboratories, Reading UK) was incubated with 10 µM BT3990 at 37°C in 50 mM MOPS, pH 7.0 overnight. The reaction was stopped by adding Phenol: Chloroform: Isopropanol (25∶24∶1), then the upper water phase was transferred to a clean Eppendorf tube and lyophilized overnight.

### SpGH38 Crystallization Data Collection and Structure Determination

Crystallization conditions for two crystal forms of recombinant SpGH38 have been established corresponding to *P*2_1_ (apo) and *P*4_3_2_1_2 (SeMet derivative and swainsonine complex) crystal systems. Both forms were obtained at 19°C in equal volumes of protein and reservoir solution. The *P*2_1_ crystal form of apo-SpGH38 was crystallized in sitting drop setup by mixing 12 mg mL^−1^ protein with 100 mM Tris, pH 8.5, 1.5 M (NH_4_)_2_SO_4_ and 12% v/v glycerol with the reservoir solution acting as the cryo-protectant. The *P*4_3_2_1_2 crystal form was obtained in hanging drop format from 15 mg mL^−1^ 3C cleaved SpGH38 mixed with 3% v/v glycerol, 54% v/v Tacsimate (pH 7.0) and 2% v/v polyethylene glycol 6000; for this form appropriate cryo-protection was afforded through increasing the Tacsimate concentration to ∼70% v/v. Crystals of the swainsonine complex form were obtained by soaking *P*4_3_2_1_2 SpGH38 crystals for ∼16 h in mother liquor supplemented with 2 mM swainsonine.

Diffraction data for SpGH38 selenomethionine derivative, apo, and swainsonine complex forms were collected at beamlines ID29-2, ID14-2, and ID14-1, respectively, of the European Synchrotron Radiation Facility. Data were processed with either the HKL2000 suite [54] or iMosflm/Scala [55], [56]. The structure of SpGH38 was solved by MAD phasing at the peak energy of 12.659 keV of a selenomethionine derivative using 0.5° oscillation for 200° and at a remote wavelength (energy 12.710 keV) for 180°. SHELXC and SHELXD [57] were used for locating selenium sites and initial density modification with autoSHARP [58]. Refinement of heavy atom positions, density modification and initial SpGH38 model building was conducted with RESOLVE [59]. A single SpGH38 molecule was then used as a molecular replacement model with the 1.9 Å native dataset in PHASER [60], followed by cycles of maximum-likelihood refinement using REFMAC [61] interspersed with manual corrections of the models using COOT [62]. Other computing used the CCP4 suite [63], unless otherwise stated. Apo and complexed structures of SpGH38 were solved by molecular replacement using PHASER [60]. Data processing and refinement statistics are presented in **Table 1**. Structural figures were drawn with PyMol (DeLano Scientific LLC).
